# Regaining Momentum in Family Planning

**DOI:** 10.9745/GHSP-D-18-00483

**Published:** 2018-12-27

**Authors:** Jose G. Rimon, Amy O. Tsui

**Affiliations:** a Bill & Melinda Gates Institute for Population and Reproductive Health, Department of Population, Family and Reproductive Health, Johns Hopkins Bloomberg School of Public Health, Baltimore, MD, USA.; b Department of Population, Family and Reproductive Health, Johns Hopkins Bloomberg School of Public Health, Baltimore, MD, USA.

## Abstract

Since the launch of the Family Planning 2020 initiative 5 years ago, 46 million more clients in the 69 poorest countries are using modern contraception—a tremendous accomplishment, albeit behind schedule to reach the 2020 global goal of 120 million. Family planning continues to be innovative, and as reflected in the recent 2018 International Conference on Family Planning in Rwanda, there is a newfound momentum behind the movement and a new generation of young leaders with powerful ideas, creativity, and passion who are stepping up to help propel family planning onward.

The 1994 International Conference on Population and Development in Cairo established that family planning should be considered a core part of reproductive health care and that women have the right to decide whether, when, and how many children to have. Yet for almost 2 decades post-Cairo, family planning's visibility receded and remained in the shadows of other global health issues, such as HIV/AIDS, malaria, and tuberculosis.^1^^,^^2^ The field, however, quietly persisted, developing new contraceptive formulations, testing approaches to expand service delivery, broadening stakeholder interest, and engaging with private-sector networks. Family planning's reemergence was assisted with the start of the series of International Conferences on Family Planning (ICFP), the first of which took place in 2009, organized by Johns Hopkins University's Bill & Melinda Gates Institute for Population and Reproductive Health and the host country government of Uganda. ICFP 2009 drew in more than 1,200 attendees, when only 300 were anticipated. The momentum of interest in family planning continued with ICFP 2011 in Senegal, attended by more than 2,200 professionals.

As the world's largest scientific and programmatic conference dedicated to family planning, ICFP brings together researchers, policy makers, ministers, advocates, practitioners, media, and youth to share knowledge and best practices. The conference takes place every 2 years, and each time is cosponsored by a different host country partner, a core group of organizers, and more than 50 agencies comprising the ICFP international steering committee. It offers a regular convening platform around which family planning stakeholders can share the latest evidence, exchange insights on best practices, and plan opportunities to network.

The International Conference on Family Planning is the world's largest scientific and programmatic conference dedicated to family planning.

The year 2012 saw the London Summit on Family Planning, a landmark gathering of the family planning movement, organized and cohosted by the Bill & Melinda Gates Foundation, the United Nations Population Fund (UNFPA), and the UK Government's Department for International Development (DFID). The London Summit gave rise to Family Planning 2020 (FP2020), an initiative which has galvanized and tracked global and national family planning commitments and achievements toward the goal of enabling 120 million more women and girls to access voluntary family planning by 2020. Total spending on family planning in the FP2020 focus countries in 2016 reached US$3.4 billion, with less than half (48%) coming from donors and one-third from domestic governments.^3^

Since 2012, ICFP 2013 was held in Ethiopia and ICFP 2016 in Indonesia, both with more than 3,000 attendees. The second London Summit followed in 2017. These events further energized global interest in and commitment to family planning. The field has recently seen the development of several technical and programmatic innovations, such as contraceptive delivery to rural areas by community health workers (task shifting) and mobile teams, access to self-administered subcutaneous Depo-Provera injectables, social franchising to harness the capacity of private provider networks, post-abortion family planning, and immediate postpartum family planning.^4^ These high-impact practices have evolved through technical consensus among implementing organizations in the family planning field and widely shared at each ICFP.

## PROGRESS IN FAMILY PLANNING GLOBALLY

The 2018 ICFP, recently held in November in Kigali, Rwanda, continues to capture and reflect the momentum of the family planning community. It was attended by more than 4,000 participants from 119 countries and the largest-ever contingent of more than 600 youth. At this latest meeting, the FP2020 progress report for 2018 was released, which noted that as of July 2017, more than 317 million women and girls in the world's 69 poorest countries were using modern contraceptives.^3^ That is an increase of 46 million since the FP2020 partnership was launched 5 years earlier and about 30% higher than the historic trend in new users. Yet the gains are not up to expectations. The report confirms the sobering reality that, although some countries may meet their individual goals, the family planning community is not on track to meet the 2020 global goal.

The 2018 conference in Kigali, Rwanda, was attended by the largest-ever contingent of more than 600 youth.

Still, the results are encouraging. Progress has been made; the curve is bending upward. There are observers in the community who believe that current achievements are underreported, that estimates are not adequately reflecting recent gains with real- and near-time data. The Indian government has expanded its provision of sponsored methods and introduced injectables and a weekly pill, as well as postpartum delivery of the intrauterine device (IUD), into the public health system. Service statistics seem to indicate an upsurge in postpartum family planning, especially with IUD use.^5^ In Nigeria, data from the Performance Monitoring and Accountability 2020 (PMA2020) project suggest that the modern contraceptive prevalence rate (mCPR) among married women was 16% in 2016 and reached 19% in 2018,^6^ a rise since the 2013 Demographic and Health Survey estimate of 10%.^7^ PMA2020 data for both Burkina Faso and Uganda show an average increase in mCPR of 2 percentage points per year over the last 3 to 4 years.^8^^,^^9^ A rapid and substantial uptick in implants in sub-Saharan Africa is also detectable; a recent study shows contraceptive implants to be the main driver of increases in mCPR in 11 sub-Saharan African countries over the last 4 to 8 years.^10^

## CRUCIAL CONTRIBUTIONS OF FAMILY PLANNING TO DEVELOPMENT GENERALLY

While the family planning field has been advancing in its efforts, it has been evident to the rest of the world that the planet needs family planning. It is a cornerstone of the Sustainable Development Goals, a key to the quest for universal health care, and a way for countries and regions to prosper. The ICFP 2018 theme, “Investing for a Lifetime of Returns,” references family planning's impressive returns on investment, in terms of the economy, education, empowerment, and the environment, not just the health benefits of reduced maternal morbidity and mortality and under-5 and infant mortality.^11^ Well-known is family planning's ability to spur economic progress in the context of the benefits of demographic dividend, which has been documented to have contributed at least 33% of the economic growth in East Asia.^12^ Family planning has also been established as the second-best “buy” for development by the Copenhagen Consensus, an independent think tank. Their research concludes that the long-term economic and health benefits of achieving universal access to contraception are worth US$120 for each $1 spent on family planning.^13^ A recent analysis found that India and Nigeria could save $89.7 billion and $12.9 billion, respectively, by satisfying current unmet need for family planning by 2030.^14^ A study led by noted environmentalist Paul Hawken estimates that investments in family planning will reduce carbon emissions by nearly 60 gigatons through 2050; family planning combined with girls' education is considered the most effective means of mitigating climate change.^15^

Family planning is a cornerstone of the Sustainable Development Goals, a key to the quest for universal health care, and a way for countries and regions to prosper.

## CONTINUED PROGRESS

While FP2020 stakeholders may not reach their goal by 2020, the needs of the remaining 73 million and more women and girls will eventually be met. This will in turn provide a strong foundation for achieving universal access to family planning by 2030. The critical question is how does one get there from here? Will the same thinking that brought the family planning community this far take it forward? Or are new ways of thinking, a fresh vision for the family planning field, needed?

In our view, there is a need to challenge current successful approaches: more “positive disruptions,” more business unusual rather than business as usual. An analysis of the international family planning movement in 2005 called out 4 possible courses of action^16^:

- Forming strategic alliances.
- Redefining the family planning message to mobilize and strengthen support.
- Improving service delivery to broaden public acceptance and contraceptive method use.
- Nurturing new leadership.

As recounted above, the first 3 have materialized since 1994. For the fourth, a breakthrough at the 2018 Kigali conference was the new generation of family planning leaders—the young people who brought powerful ideas, creativity, and passion there. They were a force to be reckoned with: they organized a sold-out youth pre-conference and a youth plenary during the main conference, made a strong showing on the scientific program and during special events, and even wrote an original new family planning song, “We are Family,” and performed it live at the closing ceremony. One youth attendee expressed a powerful idea—that ultimately, universal access will not be driven by donors or even governments but by the individual decisions of women and men, acting in their own self-interest and collectively becoming a movement, creating norms of behavior that are self-sustaining and transforming the “vicious cycle of poverty into a virtuous cycle of development.”

As one youth expressed at the International Conference on Family Planning, ultimately universal access will not be driven by donors or governments but by the individual decisions of women and men.

The future of family planning, and the planet, is already in the hands of the young people. This gives us confidence that the next decades will see amazing progress in family planning.

## Notes

**Funding:** None.
